# Supplementary material for: Metabolic Phenotypes—The Game Changer in Quality of Life of Obese Patients?
Source: Healthcare (Basel). 2022 Mar 25;10(4):617. doi: 10.3390/healthcare10040617 (PMC9025564; doi:10.3390/healthcare10040617)
Supplement: Supplementary file 1 [file healthcare-10-00617-s001.zip › healthcare-1637971 Supplimentary material.pdf]

Table S1. Normality tests for quality of life scales and scores.

	Skewness	Kurtosis	Kolmogorov-Smirnov (p)	Shapiro–Wilk (p)
PF	−0.258	−0.998	0.001	0.002
RP	0.029	−1.655	0.000	0.000
BP	0.097	−0.663	0.000	0.000
GH	−0.028	−0.643	0.073	0.163
V	−0.067	−0.378	0.051	0.207
SF	−0.240	−1.057	0.000	0.000
RE	−0.263	−1.703	0.000	0.000
MH	−0.745	0.650	0.045	0.002
PCS	0.027	−0.725	0.200	0.198
